# Therapeutic Potential of Extracellular Vesicles in Oral Inflammation

**DOI:** 10.3390/ijms26073031

**Published:** 2025-03-26

**Authors:** Yan Rou Farm, Bing Huan Chuah, Jia Xian Law, Xin Fang Leong, Masfueh Razali, Sook Luan Ng

**Affiliations:** 1 Department of Craniofacial Diagnostics and Biosciences, Faculty of Dentistry, Universiti Kebangsaan Malaysia, Kuala Lumpur 50300, Malaysia; p145608@siswa.ukm.edu.my (Y.R.F.); p140783@siswa.ukm.edu.my (B.H.C.); leongxinfang@ukm.edu.my (X.F.L.); 2 Department of Tissue Engineering and Regenerative Medicine, Faculty of Medicine, Universiti Kebangsaan Malaysia, Kuala Lumpur 56000, Malaysia; lawjx@hctm.ukm.edu.my; 3 Department of Restorative Dentistry, Faculty of Dentistry, Universiti Kebangsaan Malaysia, Kuala Lumpur 50300, Malaysia; masfuah@ukm.edu.my

**Keywords:** periodontitis, extracellular vesicles, inflammation

## Abstract

The therapeutic potential of extracellular vesicles (EVs) in reducing oral inflammation is thoroughly examined in this review, with an emphasis on gingivitis, periodontitis, and oral mucositis. It explains the complex relationship between microbial dysbiosis and host immune responses in the aetiology of oral inflammation. Pathophysiological mechanisms of periodontitis are examined, emphasising the roles played by periodontal pathogens and inflammatory mediators in the disease’s chronic course and systemic effects. Preclinical research is providing new evidence that EVs originating from various cellular sources control immune cell dynamics towards a pro-healing phenotype, promote tissue regeneration, and have immunomodulatory qualities. EV-based therapies appear to be a promising new therapeutic technique with potential benefits over traditional methods for the treatment of oral inflammatory illnesses by specifically altering inflammatory signalling pathways. This review highlights the potential of EVs to improve patient outcomes in oral health and emphasises the need for additional clinical research to clarify the therapeutic efficacy and underlying mechanisms of EVs in periodontal therapy.

## 1. Introduction

### 1.1. Overview of Oral Inflammation

Oral inflammation encompasses conditions such as gingivitis, periodontitis, and oral mucositis, all of which present symptoms like discomfort, bleeding, swelling, and redness. The underlying causes of these conditions are multifactorial, involving microbial infections, immune responses, and systemic health disorders [1]. A bacterial polymicrobial biofilm on the teeth can lead to gingivitis, a common inflammatory oral situation [2]. This biofilm can increase the likelihood of developing periodontitis, a more severe and destructive periodontal disease. Additionally, it elevates inflammatory biomarkers, contributing to tooth loss, aesthetic concerns, impaired oral function, and a diminished quality of life [2]. For effective periodontal diagnosis and prognosis, it is essential to identify periodontal pathogen by-products, such as genomic DNA (gDNA), within salivary small extracellular vesicle (sEV) populations [3]. Given the inflammatory nature of periodontal diseases, gingivitis is also linked to increased levels of salivary inflammatory cytokines, including IL-6, TNF-α, IL-1β, IL-8, and IL-10 [3]. Chronic inflammation caused by an imbalance in the subgingival biofilm is a hallmark of periodontitis, which can lead to tooth loss if treatment is not received [4]. *Porphyromonas gingivalis* and *Aggregatibacter actinomycetemcomitans* are two periodontal infections associated with this illness that cause tissue damage by inducing inflammatory and immunological responses [5,6]. In addition, periodontitis causes the secretion of cytokines such as TNF-α, IL-1β, and IL-6, leading to systemic inflammation, which may connect periodontal disease to a number of systemic problems, such as cardiovascular and neurological conditions [5,7]. Elevated myelopoietic activity, persistent low-grade inflammation, and bacteremia are key contributors to periodontitis, and recent studies have shown that periodontopathogen-produced bacterial extracellular vesicles (bEVs) can penetrate the blood–brain barrier and induce neuroinflammation, potentially contributing to diseases like Alzheimer’s [8,9]. A mismatch between matrix metalloproteinases and their inhibitors during active periodontal disease also exacerbates additional tissue damage, which intensifies the inflammatory response [10]. This review aims to provide an in-depth analysis of the therapeutic potential of extracellular vesicles (EVs) in reducing oral inflammation, with particular emphasis on gingivitis, periodontitis, and oral mucositis. Specifically, we examine how EVs modulate the host immune response, promote tissue regeneration, and serve as diagnostic biomarkers in the oral cavity. By highlighting recent preclinical findings and discussing current challenges, we seek to clarify the underlying mechanisms through which EVs exert their effects, thereby guiding future clinical research and the development of EV-based therapies.

### 1.2. EVs: Definition and Classification

EVs are lipid bilayer-enclosed particles secreted by nearly all cell types that serve as essential mediators of intercellular communication by transferring proteins, lipids, and nucleic acids between cells [11,12]. Based on their biogenesis and size, EVs are commonly classified into three main subtypes:

Exosomes:

Exosomes are typically 30–150 nm in diameter and are formed as intraluminal vesicles (ILVs) within multivesicular bodies (MVBs). When MVBs fuse with the plasma membrane, these ILVs are released as exosomes. They are generally enriched in proteins associated with the endosomal pathway, such as tetraspanins CD9, CD63, and CD81, as well as TSG101 and ALIX, which serve as standard markers for exosomal identification [11,12,13].

Microvesicles:

Microvesicles (also known as ectosomes or microparticles) range in size from approximately 100 to 1000 nm and are produced directly by the outward budding of the plasma membrane. These vesicles frequently expose phosphatidylserine on their outer surface and often contain proteins reflective of their parent cell’s plasma membrane [14]. Due to overlaps in size and composition with exosomes, many researchers now refer to vesicles under 200 nm as “small EVs (sEVs)” when their precise origin is not determined [12].

Apoptotic Bodies:

Apoptotic bodies are larger vesicles (typically 1–5 μm) that are released by cells undergoing apoptosis [15]. They often encapsulate cellular fragments such as genomic DNA and histones and are generally cleared rapidly by phagocytes [16].

Among these EV subtypes, human platelet-derived extracellular vesicles (hPLT-EVs) are of particular interest. Platelets secrete EVs, including both exosomes and microvesicles (MVs), in response to activation and apoptosis or during storage; these vesicles comprise a major fraction of circulating EVs in blood [17]. hPLT-EVs, including both microvesicles and exosomes, are released into the circulatory system in significant quantities by platelets, spanning a broad size range from approximately 50 to 1500 nm [12]. These EVs can be taken up by recipient cells, initiating specific cellular responses [15]. Platelet-derived exosomes, a subset of hPLT-EVs, are small vesicles enclosed within multivesicular bodies, whereas membrane-derived vesicles, previously known as microparticles, generally fall into the medium-to-large size range and play a key role in intercellular communication by delivering bioactive compounds [16]. hPLT-EVs not only display the common exosomal markers (CD9, CD63, and CD81) but also express platelet-specific surface proteins such as CD41, CD42, and CD62P, which help to distinguish them from EVs originating from other cell types [14,17]. In addition to their established role in haemostasis, platelet-derived EVs have been implicated in immune modulation, angiogenesis, tissue repair, and even cancer progression [13,17]. Their natural origin confers advantages including high biocompatibility, low immunogenicity, and the ability to cross biological barriers, making them promising vehicles for drug delivery and regenerative medicine applications [13,17]. Early studies estimated that membrane-derived vesicles comprised 70–80% of all microvesicles; however, a recent freeze-fixation electron microscopic investigation of CD41-positive vesicles in plasma led to the revision of this estimate to about 25% [18]. It is also suggested that megakaryocytes, rather than platelets, may be the primary source of CD41-positive EVs in circulation. Conversely, elevated platelet activation, particularly under conditions such as thrombosis or inflammation, is associated with an increased release of platelet-derived EVs [18].

### 1.3. Search Strategy

We conducted electronic searches in databases including PubMed, Scopus, and Web of Science for peer-reviewed articles published in English from 2000 to the present. Key terms used in the search were “extracellular vesicles”, “exosomes”, “microvesicles”, “oral inflammation”, “periodontitis”, “gingivitis”, and “oral mucositis”, along with related terms. Studies were included if they provided insights into EV biogenesis, composition, or function and focused on their roles in modulating oral inflammation, promoting tissue regeneration, or serving as diagnostic biomarkers in oral health. Both original research articles and review papers that presented novel data or comprehensive syntheses were considered. Articles that were not peer-reviewed, not published in English, or not directly relevant to oral inflammatory conditions were excluded. Full texts of selected studies were then reviewed, and reference lists were examined to identify additional pertinent publications.

## 2. Mechanisms of Inflammation in the Oral Environment

### 2.1. Role of the Immune System in Oral Inflammation

Periodontitis arises from intricate cellular and molecular interactions, where tissue damage occurs due to an amplified immune response triggered by microbial imbalance [19]. Key cellular players include T and B lymphocytes, osteoclasts, neutrophils, and macrophages. Neutrophils, the first line of defence, release antimicrobial peptides and reactive oxygen species, although excessive neutrophil activity can harm tissues [20]. Macrophages have dual roles: M1 macrophages induce tissue damage and inflammation, whereas M2 macrophages aid in tissue repair [20]. Regulatory T cells (Tregs) help control overactive immune responses, while T-helper cells, particularly Th17, enhance inflammation by producing IL-17 [21]. The innate immune system, which comprises these immune cells, along with mucosal barriers such as saliva (containing antimicrobial proteins like lysozyme and defensins) and pattern recognition receptors like Toll-like receptors (TLRs) that detect microbial components, is critical in initiating inflammatory responses and maintaining oral health [20,22,23,24,25]. Persistent imbalance in these mechanisms accelerates periodontitis progression and further tissue destruction. Table 1 summarises these key components of the innate immune system and their functions.

If innate immunity is insufficient, the adaptive immune system is activated to provide a more specific response (Table 2).

Managing oral inflammation often involves targeting the immune system to reduce excessive responses while preserving protective immunity. Therapeutic strategies are included in Table 3.

### 2.2. Cellular and Molecular Pathways in Periodontitis

At the molecular level, several signalling pathways regulate the inflammatory response in periodontitis. The Toll-like receptor (TLR) pathway, upon detecting bacterial components, activates nuclear factor kappa B (NF-κB), leading to cytokine release [27]. The mitogen-activated protein kinase (MAPK) pathway not only stimulates bone resorption but also plays a role in osteoclast development and cytokine production [20]. Additionally, the receptor activator of the nuclear factor kappa-B ligand (RANKL) pathway is essential for osteoclast activation, contributing to alveolar bone loss [4]. Disruption of these pathways results in a chronic inflammatory environment, exacerbating the disease and causing further tissue destruction [28].

### 2.3. Current Anti-Inflammatory Strategies in Oral Disease

Many anti-inflammatory approaches that target important immunological and biochemical pathways have been developed to treat oral disorders. To reduce inflammation and stop tissue damage, pharmacological therapies such as corticosteroids and nonsteroidal anti-inflammatory medications (NSAIDs) are frequently employed [19,22]. Oral inflammation may be controlled by biologic treatments, such as monoclonal antibodies that target pro-inflammatory cytokines, including TNF-α and IL-17.

Novel nanotechnology-based strategies, such as drug delivery systems utilizing nanoparticles, have surfaced to increase the efficacy of anti-inflammatory drugs while reducing adverse effects [29]. Furthermore, the potential of probiotic treatments to alter immune responses and reduce inflammation has been studied. These treatments seek to restore the microbial balance in the mouth [30]. Natural and herbal substances with anti-inflammatory qualities, such as flavonoids and polyphenols, are being researched as potential additions to conventional therapies [31].

**Figure 1:**
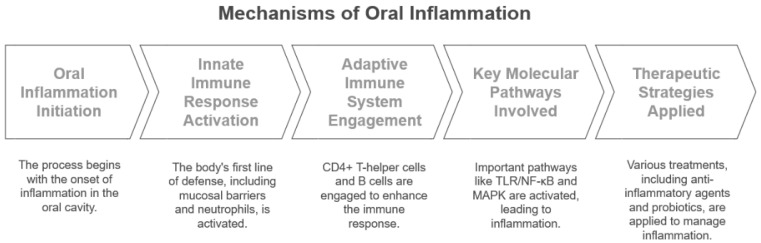
Mechanisms of oral inflammation and therapeutic strategies. The mechanism of oral inflammation involves initiation, immune activation, and key molecular pathways. Various therapeutic strategies have been applied to manage inflammation.

The mechanisms underlying oral inflammation and corresponding anti-inflammatory strategies are summarised in Figure 1.

## 3. EVs: Characteristics and Therapeutic Potential

### 3.1. Biogenesis, Composition, and Functional Roles of EVs

EVs are membrane-bound nanoparticles released by a variety of cell types that play a pivotal role in intercellular communication. They are formed via distinct mechanisms; exosomes are generated as intraluminal vesicles (ILVs) within multivesicular bodies (MVBs) and typically measure 40–100 nm in diameter, while MVs (also known as ectosomes or microparticles) are shed directly from the plasma membrane and generally range from 50 to 1000 nm [10,31]. The composition of EVs reflects their cellular origin; for example, exosomes are enriched in tetraspanins such as CD9, CD63, and CD81, as well as proteins involved in endosomal sorting like TSG101 and ALIX, and they are also characterised by specific lipid profiles including high levels of cholesterol and sphingomyelin [32,33]. In contrast, MVs often expose phosphatidylserine on their outer surface and include proteins representative of the plasma membrane [32].

Beyond serving as biomarkers, the ability of EVs to transport bioactive molecules, including proteins, lipids, and various forms of RNA (mRNA, microRNA, etc.), allows them to modulate key biological processes such as inflammation, immune response, tis-sue regeneration, and cell signalling [32]. Their functional involvement in both protective and pathological responses (e.g., tissue healing versus cancer progression) underpins their emerging role in therapeutic applications [11,13,32]. Because EVs selectively package their cargo in a manner that reflects the state of the parent cell, they also offer a “molecular fingerprint” that can be exploited for diagnostic purposes [33,34].

### 3.2. Sources of EVs for Therapeutic Applications

Because EVs inherently regulate immune responses, promote tissue regeneration, and facilitate intercellular communication, they represent promising therapeutic agents. EVs can be isolated from various cell sources, each offering unique advantages for clinical use. For example, mesenchymal stem cell (MSC)-derived EVs promote tissue repair and modulate immune responses [19], platelet-derived EVs enhance wound healing and support vascular regeneration [35], immune cell-derived EVs help modulate cytokine production and facilitate antigen presentation [36]. Epithelial cell-derived EVs influence host–microbiome interactions and oral health [28], and EVs from other stem cell sources exhibit regenerative properties similar to MSC-derived EVs but may face ethical and production challenges [21]. Furthermore, synthetic and engineered EVs are being developed to achieve targeted drug delivery and improved treatment efficacy [35]. Table 4 summarises the key cell sources of EVs, their functions, and relevant citations.

### 3.3. Mechanisms of EV Action in Modulating Inflammation

EVs are increasingly important in the regulation of inflammation and the immune system. Numerous cell types, including immune cells, epithelial cells, and mesenchymal stem cells, release EVs, which are essential for immunological control and cell-to-cell communication [29]. They transport bioactive substances including proteins, lipids, and nucleic acids that affect inflammatory and immunological responses [35].

One primary way EVs reduce inflammation is by suppressing pro-inflammatory cytokines. For example, MSC-derived EVs can lower levels of TNF-α, IL-6, and IL-1β, thereby decreasing excessive immune activity [37]. Additionally, EVs help polarise macrophages to an anti-inflammatory M2 type, reducing inflammation and aiding in tissue repair.

EVs play a crucial role in adaptive immunity by influencing T-cell responses. They regulate T-helper cell differentiation and expand T-regulatory cells, helping to maintain immune balance [35]. Additionally, epithelial cell-derived EVs contain microRNAs that can suppress NF-κB c, reducing chronic inflammation in periodontal disease

Another significant function of EVs is in tissue regeneration. Stem cell-derived EVs carry regenerative factors that promote fibroblast proliferation, extracellular matrix remodelling, and angiogenesis, which aid in periodontal tissue repair. Furthermore, EVs can impact osteoclast differentiation by modulating the RANK, RANKL, and osteoprotegerin (OPG) pathway, thereby protecting against bone loss seen in periodontitis.

**Figure 2:**
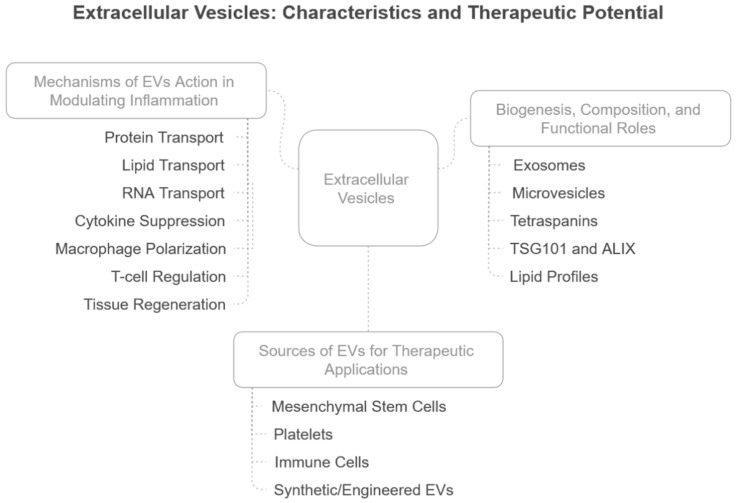
Characteristics and therapeutic roles of EVs. The characteristics of EVs include their biogenesis, composition, and functional roles. Various sources of EVs for therapeutic applications and their mechanisms in modulating inflammation are highlighted.

The characteristics of EVs and their mechanisms of inflammation modulation are summarised in Figure 2.

## 4. Evidence of EV Therapeutics in Oral Inflammation

The ability of EVs to regulate oral inflammatory processes is demonstrated by recent research. EVs from a variety of origins, including mesenchymal stem cells and oral cancer cells, have shown promise in promoting tissue regeneration, altering the microenvironment, and influencing immune cell behaviour in experimental models of periodontitis and gingivitis [38]. Comparative studies suggest that by focusing on inflammatory pathways, EV-based therapies may be superior to traditional ones [39,40].

### 4.1. EVs in Periodontitis: Experimental Studies

EVs can significantly influence the inflammatory environment associated with periodontitis. In a ligature-induced periodontitis model, Nakao and research team members demonstrated that exosomes derived from TNF-α-preconditioned gingival mesenchymal stem cells (GMSCs) reduced the local inflammatory response [41]. This was evidenced by a decrease in pro-inflammatory cytokine levels and a reduction in TRAP-positive osteoclasts [24]. Similarly, Wang et al. (2024) found that exosomes produced by periodontal ligament stem cells (PDLSCs) inhibited the release of pro-inflammatory mediators in vitro while also increasing osteogenic markers such as BMP-2 and RUNX2, thereby promoting tissue regeneration [42]. These findings highlight the potential of EVs derived from PDLSCs and GMSCs as a novel cell-free therapeutic approach for periodontal regeneration due to their dual ability to reduce inflammation and promote regenerative processes in periodontitis.

Several independent studies have provided additional evidence that EVs can modulate immune responses by favouring anti-inflammatory M2 macrophage polarisation. For instance, Wang et al. (2020) showed that mesenchymal stem cell-derived EVs are enriched in miR-146a, which can inhibit NF-κB signalling in recipient macrophages [43]. This shifts their phenotype toward M2 polarisation, reducing inflammatory cytokine production and promoting tissue repair [44]. In another study, Hou et al. (2023) [45] discovered that EVs from human umbilical cord mesenchymal stem cells contained significant levels of miR-223. When taken up by macrophages, these EVs downregulated key inflammatory mediators in the NF-κB pathway and induced M2-like polarisation. This contributed to an anti-inflammatory microenvironment and enhanced healing in a murine model of myocardial infarction [45].

These studies support the idea that the anti-inflammatory cargo of EVs, including miRNAs such as miR-146a and miR-223, plays a crucial role in dampening NF-κB signalling, promoting M2 macrophage polarisation and ultimately facilitating tissue healing.

### 4.2. EVs in Gingivitis and Other Oral Inflammatory Conditions

EVs are increasingly acknowledged as key players in various oral inflammatory diseases beyond periodontitis. In the context of oral cancer, research indicates that EVs from cancer cells can transform nearby fibroblasts into cancer-associated fibroblasts (CAFs), thereby altering the tumour microenvironment. For example, Arebro et al. (2023) [46] demonstrated that EVs from oral squamous cell carcinoma (OSCC) activate CAFs, leading to elevated levels of pro-inflammatory cytokines like CXCL5 and IL-8; these activated CAFs subsequently secrete additional cytokines, exacerbating local inflammation and promoting tumour progression [47]. Similarly, Dourado et al. (2019) showed that OSCC cells internalise CAF-derived EVs in vitro, activating NF-κB and MAPK pathways and resulting in an inflammatory secretome [36,46]. In addition, Qin et al. (2019) reported that EV-mediated microRNA transfer alters gene expression in recipient fibroblasts, accelerating their conversion into pro-inflammatory CAFs and creating a microenvironment rich in IL-8, which recruits neutrophils and sustains inflammatory responses [48,49]. Gene set enrichment analyses (GSEAs) further reveal that cancer cell-derived EVs can reprogram neighbouring fibroblasts by upregulating the TNFα and IL6/JAK/STAT3 signalling pathways, diverging from the classical TGFβ-induced activation typically associated with myofibroblastic differentiation [47]. This inflammatory activation suggests that EVs not only modulate the tumour microenvironment but also offer potential as biomarkers and therapeutic targets in conditions such as oral lichen planus.

Emerging evidence also indicates that EVs significantly influence the oral inflammatory environment in non-malignant conditions like gingivitis and other mucosal inflammatory disorders. Cai et al. (2023) reported that EVs derived from gingival crevicular fluid (GCF) in patients with periodontitis are significantly increased and enriched with pro-inflammatory cytokines [50,51]. These mediators disrupt gingival epithelial integrity, facilitating immune cell infiltration into the subepithelial tissue and thereby exacerbating local inflammation [51]. Furthermore, EV-mediated signalling in CAFs is associated with elevated expression of pro-inflammatory chemokines and cytokines, with particular involvement of the IL6/JAK/STAT3 axis in disease progression [52].

### 4.3. Comparative Studies of EVs and Conventional Therapies

Comparative studies have begun evaluating the efficacy of EV-based therapies against conventional anti-inflammatory treatments. For example, research has shown that EVs derived from gingival mesenchymal stem cells have strong anti-inflammatory effects in models of periodontal inflammation [53].

In one study, GMSC-derived exosomes were applied to inflamed periodontal tissues in a rat bone defect model, leading to a significant increase in osteogenic markers (RUNX2, VEGFA, OPN, and COL1A1) and substantial osteogenic regeneration, including enhanced bone formation and blood vessels [54]. These findings suggest that EV-based interventions not only effectively reduce inflammation but also help restore tissue homeostasis without the long-term side effects associated with systemic corticosteroids or NSAIDs [40]. Moreover, EVs can be engineered or preconditioned to enhance their therapeutic cargo further, making them a promising platform for targeted anti-inflammatory therapy, leading to safer and more effective treatments for chronic inflammatory diseases [40].

Additionally, EVs offer the advantage of targeted delivery, as they tend to accumulate in inflamed tissues. A study demonstrated that MSCs derived from human GMSCs regenerated lost epithelial lining in mouse models of oral mucositis [55]. Similarly, a study reported that EVs were internalised by inflamed oral tissues through receptor-mediated mechanisms, effectively concentrating their therapeutic cargo at the site of inflammation [56].

## 5. Human Platelet-Derived EVs in Periodontitis

The role of hPLT-EVs as important mediators in tissue regeneration and inflammatory regulation is becoming more widely acknowledged. These vesicles show promise in treating periodontitis.

### 5.1. Extraction and Chararcterisation of hPLT-EVs

hPLT-EVs have shown great promise as a cell -ree therapeutic platform because of their potent biological activity and regenerative capabilities. hPLT-EVs can be isolated from human platelet lysate using methods that preserve both vesicle integrity and bioactivity. For example, size exclusion chromatography (SEC) is an effective technique for separating EVs from protein contaminants and larger vesicular structures while maintaining vesicle functionality [57]. In a comparative study, SEC was shown to yield purer EV preparations than conventional ultracentrifugation while better preserving bioactivity, which is crucial for clinical applications [58]. Therefore, standardised, scalable isolation methods like SEC are critical in achieving reproducible yields and high-quality EV preparations that meet regulatory standards [59].

Characterisation of hPLT-EVs is typically performed using several complementary techniques. Nanoparticle tracking analysis (NTA) is used to assess particle size distribution and concentration. For instance, hPLT-EVs isolated from human platelet-rich plasma had a mean particle diameter of approximately 175 to 246 nm, with a narrow size distribution, supporting the reproducibility and consistency of the isolation protocol [60]. Western blot analysis is routinely used to verify the expression of canonical exosomal markers such as CD9 and CD63, confirming the identity of these vesicles as exosomes and validating the isolation process. This approach aligns with the guidelines provided by the Minimal Information for Studies of Extracellular Vesicles 2023 (MISEV2023), which recommend combining quantitative techniques like NTA with biochemical methods such as immunoblotting for robust EV characterisation.

Additionally, electron microscopy (EM) is commonly used to assess the morphology of hPLT-EVs. Transmission electron microscopy (TEM) shows that hPLT-EVs exhibit a characteristic morphology with a clearly defined lipid bilayer [61]. Similarly, cryo-electron microscopy confirms that these hPLT-EVs maintain an intact bilayer membrane, ensuring that the vesicles retained their functional morphology for downstream biological applications [62].

### 5.2. Anti-Inflammatory Properties of hPLT-EVs

Recent research has been increasingly focusing on the use of the anti-inflammatory and regenerative properties of hPLT-EVs as a cell-free therapeutic strategy for periodontitis. In periodontitis, chronic inflammation results in the progressive destruction of periodontal tissues, making it crucial to balance pro- and anti-inflammatory signals for effective healing. For instance, it was demonstrated that hPLT-EVs significantly enhance wound healing in vitro by promoting the migration and proliferation of gingival fibroblasts and oral keratinocytes, which are key players in periodontal tissue repair [57]. Treatment with hPLT-EVs led to faster closure of wound gaps in scratch assays, upregulation of extracellular matrix components, and reductions in pro-inflammatory cytokine levels, creating a microenvironment conducive to tissue regeneration.

Additionally, the therapeutic benefits of hPLT-EVs extend beyond wound closure. An article by Sun et al. (2022) discussed how these vesicles modulate inflammatory responses by delivering bioactive molecules such as growth factors and cytokines, which not only suppress inflammation but also stimulate angiogenesis [63]. Angiogenesis is essential in the supply of nutrients and oxygen to regenerating tissues. This dual action of reducing inflammatory mediators and promoting cell proliferation is particularly beneficial for periodontal regeneration.

Moreover, hPLT-EVs can enhance the expression of genes involved in tissue remodelling and repair, including collagen type I and fibronectin, reinforcing their role in restoring periodontal structure [64]. Thus, hPLT-EVs present a novel therapeutic tool for periodontitis, offering potential advantages over conventional treatments by simultaneously mitigating inflammation and promoting tissue regeneration.

hPLT-EVs carry diverse cargo, including various growth factors, anti-inflammatory cytokines, and specific microRNAs (miRNAs) that coordinate local immune regulation. Studies have shown that miR-223 and miR-146a, commonly found in platelet-derived EVs, are crucial in reducing inflammatory reactions. For instance, it was demonstrated that miR-223 inhibits the production of key mediators in pro-inflammatory signalling pathways, such as components of the NF-κB pathway, which is essential for the transcriptional control of numerous pro-inflammatory cytokines [65]. Similarly, miR-146a targets and reduces the expression of TNF receptor-associated factor 6 (TRAF6) and interleukin-1 receptor-associated kinase 1 (IRAK1), further inhibiting NF-κB activation and promoting macrophage polarisation toward the M2 (anti-inflammatory) phenotype [66].

Although direct studies on the effects of hPLT-EVs in periodontal settings are still in their early stages, preliminary in vitro data suggest that these vesicles can attenuate local inflammation by reducing the secretion of pro-inflammatory mediators and enhancing the presence of M2-polarised macrophages [42]. This shift in the local immune environment is crucial during periodontal healing, as excessive inflammation can impair tissue repair and regeneration [42]. Although direct studies on the effects of hPLT-EVs in periodontal settings are still in their early stages, preliminary in vitro data suggest that these vesicles can attenuate local inflammation by reducing the secretion of pro-inflammatory mediators and enhancing the presence of M2-polarised macrophages [42,54]. This shift in the local immune environment is crucial during periodontal healing, as excessive inflammation can impair tissue repair and regeneration [43].

### 5.3. Preclinical Evidence in Ligature-Induce Periodontitis Models: In Vitro and In Vivo Studies

Preclinical animal studies have highlighted the promising therapeutic potential of hPLT-EVs in treating periodontitis. In a ligature-induced periodontitis model, which closely mimics human periodontal inflammation and tissue destruction [67], local delivery of hPLT-EVs encapsulated within a hyaluronic acid (HA) gel significantly improved periodontal healing. Antich-Rosselló et al. (2022) found that applying HA-EV formulations to bone defect sites around titanium implants in rabbits resulted in significant reductions in markers of tissue necrosis and immature bone, such as lactate dehydrogenase (LDH) and alkaline phosphatase (ALP) activity [57]. This also led to a decreased fibrotic response, suggesting that hPLT-EVs can create a more favourable environment for bone regeneration and implant integration.

In addition to these in vivo findings, in vitro studies also support the regenerative capabilities of hPLT-EVs. Research on gingival fibroblasts and keratinocytes has shown that treatment with hPLT-EVs enhances cell migration and proliferation, which are key processes in wound closure and soft tissue healing [63]. These cellular effects are essential for re-epithelialisation and the restoration of periodontal tissue integrity, helping to mitigate the progression of periodontitis [63].

Recent research has reviewed the applications of hPLT-EVs in soft tissue regeneration, highlighting their potential in modulating inflammatory responses and promoting osteogenic differentiation of mesenchymal cells [63]. The study found that EVs derived from platelet lysate reduce pro-inflammatory cytokine release and enhance neovascularisation at defect sites, which is crucial for supplying oxygen and nutrients to support new tissue formation.

Further evidence suggests that combining hPLT-EVs with biocompatible carriers such as hyaluronic acid (HA) gels improve the retention and local release of EVs while also preserving their bioactivity in inflammatory microenvironments like those seen in periodontitis [68]. This synergistic approach has been shown to promote the deposition of mature bone matrix and reduce local inflammatory markers, even though it did not increase bone-to-implant contact [36,57]. Nonetheless, these findings indicate that hPLT-EVs have the potential to improve clinical outcomes for periodontal regeneration by creating a favourable microenvironment for tissue repair and reducing inflammation.

**Figure 3:**
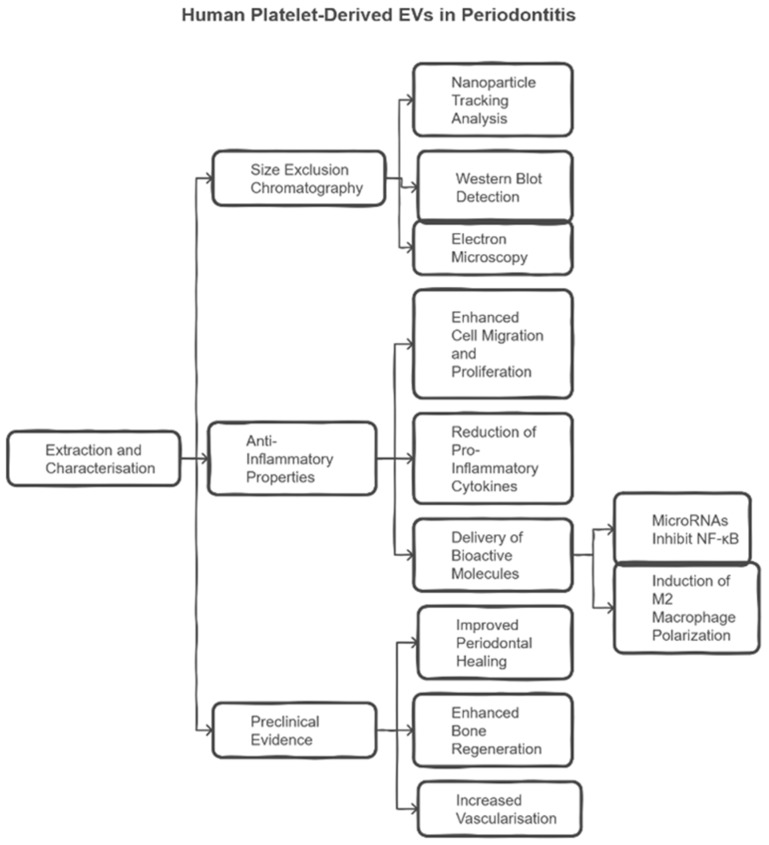
Characterisation, anti-inflammatory properties, and preclinical evidence of hPLT-EVs in periodontitis treatment. EV characterisation methods include nanoparticle tracking analysis, Western blot analysis, and electron microscopy. hPLT-EVs promote cell migration and proliferation, reduce pro-inflammatory cytokines, and facilitate the delivery of bioactive molecules. Preclinical evidence supports their role in improving periodontal healing, bone regeneration, and vascularisation.

The characterisation parameters of hPLT-EVs, their anti-inflammatory properties, and preclinical evidence supporting their use in periodontitis treatment are summarised in Figure 3.

## 6. Advantages and Challenges of EV-Based Therapies

The ability of EVs to transport bioactive cargo (proteins, lipids, and nucleic acids) between cells has garnered significant interest as a novel therapeutic modality in immunomodulation and regenerative medicine. This ability can impact immune responses and tissue repair [68]. Reduced immunogenicity, targeted distribution, and the potential to pass beyond biological barriers are just a few advantages that these vesicles have over traditional treatments [58].

However, significant challenges remain in their clinical translation. For example, current manufacturing methods yield heterogeneous EV populations, and scalable production platforms are still under development [69]. Moreover, the absence of universally accepted isolation and characterisation protocols complicates quality control, while regulatory agencies continue to refine frameworks to address the unique properties of EV-based products [70].

### 6.1. Benefits of EVs over Traditional Treatments

EVs offer several key advantages compared to traditional therapies, including cell-based treatments and small-molecule drugs [71]:

#### 6.1.1. Biocompatibility and Low Immunogenicity

EVs are naturally secreted by virtually all cell types, which makes them highly biocompatible [36,58]. Because they originate from endogenous cellular processes, they are generally recognised as self by the host immune system and exhibit a low immunogenic profile [72]. This contrasts with whole-cell therapies, where intact donor cells express a wide array of surface antigens such as major histocompatibility complex (MHC) molecules, which can provoke immune rejection and adverse inflammatory responses [71]. In several preclinical studies, EVs derived from mesenchymal stem cells (MSCs) have demonstrated minimal immune activation in vitro and in vivo, further supporting their use as safe drug delivery vehicles [73].

Furthermore, EVs’ inherent makeup enables them to avoid the mononuclear phagocyte system’s quick clearance [74]. Compared to many synthetic nanoparticles and cell-based therapies, their extended circulation period improves the transport efficiency of therapeutic payloads and reduces the need for repeated doses [75]. Remarkably, no appreciable immunotoxicity has been noted, even in cross-species applications, such as the use of EVs produced from bovine milk in mouse models, underscoring their universal biocompatibility [76]. 

#### 6.1.2. Targeted Delivery and Enhanced Stability

By shielding their internal cargo from enzymatic degradation, EVs’ distinctive lipid bilayer provides exceptional durability and a flexible therapeutic delivery platform [77]. When circulating in biological fluids, this membrane, which is composed of ceramide, sphingomyelin, and cholesterol, helps preserve the integrity of therapeutic molecules, such as proteins, lipids, and RNAs, increasing their bioavailability and extending their half-life in vivo [78].

EVs can be made to display specific targeted ligands on their exterior [71]. It was demonstrated that exosomes coated with a rabies virus glycoprotein (RVG) peptide could effectively cross the blood–brain barrier and transfer siRNA to neural cells [79]. It was shown that modified EVs might target oncogenic KRAS in pancreatic cancer, highlighting the potential for surface modifications to enhance targeting specificity [80]. These changes not only make it easier to distribute medication precisely to diseased tissues, but they also reduce off-target effects, which is important in achieving improved treatment results [81].

#### 6.1.3. Ability to Cross Biological Barriers

EVs possess the intrinsic ability to cross biological barriers [71]. For instance, it was demonstrated that multiple exosome populations can cross the blood–brain barrier (BBB), with influx rates ranging from 0.044 to 0.524 µL/g-min, highlighting their ability to move from the bloodstream into the brain parenchyma via transcytotic pathways [82]. Similarly, an in vitro BBB model was used to provide visual evidence that EVs are internalised by brain endothelial cells through receptor-mediated endocytosis and subsequently trafficked across the cell layer, suggesting that EVs utilise active transcytosis rather than passive diffusion [83].

In addition to crossing the BBB, EVs have also been observed to traverse mucosal membranes. It was reported that intranasally administered EVs loaded with anti-inflammatory compounds efficiently reached the brain, likely bypassing the mucosal barrier via the olfactory and trigeminal nerve pathways [84].

#### 6.1.4. Cell-Free Therapy

EVs have shown great promise as a cell-free alternative to traditional stem cell transplantation, bypassing critical safety concerns such as tumorigenicity and uncontrolled differentiation. EVs isolated from bone marrow–derived mesenchymal stem cells (BM-MSCs) can stimulate periodontal ligament cell proliferation and migration in vitro [85]. When administered locally in a rat periodontal defect model, these EVs significantly enhanced the regeneration of periodontal tissues by increasing new cementum deposition and reducing inflammatory cytokine expression.

Similarly, exosomes derived from stem cells from human exfoliated deciduous teeth (SHED) promoted angiogenic gene expression in endothelial cells [86]. They also increased new bone formation within periodontal defects through the activation of the AMPK pathway and TGF-β/SMAD2/3 signalling, demonstrating the multifaceted regenerative potential of EVs.

As acellular entities, EVs transport their therapeutic cargo without replicating or differentiating, eliminating the risks of uncontrolled cell growth and aberrant differentiation associated with live cell therapies [71]. This makes EV-based approaches particularly attractive for regenerative therapies such as periodontal regeneration and tissue repair. Moreover, EVs can be manufactured under standardised, scalable conditions and stored more easily than living cells, further enhancing their appeal as a safe and practical therapeutic modality [87].

#### 6.1.5. Intrinsic Cargo Diversity

EVs carry a diverse array of bioactive substances, reflecting the physiological state of their donor cells, including proteins, lipids, messenger RNAs, and microRNAs [88]. Besides that, EVs derived from mesenchymal stem cells contain specific microRNAs and proteins that promote macrophage polarisation toward an anti-inflammatory phenotype, thereby modulating immune responses.

In another study, it was found that EVs isolated from hypoxia-preconditioned MSCs were enriched with angiogenic factors such as vascular endothelial growth factor (VEGF) and erythropoietin, enhancing neovascularisation and tissue repair in a hindlimb ischemia model [19]. Similarly, EVs from activated immune cells have been shown to contain cytokines and regulatory microRNAs that downregulate pro-inflammatory signalling in recipient cells, coordinating an immune response that supports tissue regeneration.

These findings demonstrate that the natural cargo of EVs offers a multifaceted therapeutic approach. EVs can modulate immune responses, promote angiogenesis, and stimulate tissue repair, achieving outcomes that are difficult to attain with single-molecule drugs [89].

### 6.2. Challenges in EV Manufacturing and Standardisation

Despite their therapeutic promise, several technical challenges hinder the translation of EV-based therapies:

#### 6.2.1. Isolation and Purification

The harnessing of EV therapies faces several technical challenges that must be addressed for successful translation from the bench to the clinic. One critical step is the isolation and purification of EVs. Common methods like ultracentrifugation, size exclusion chromatography (SEC), and immunoaffinity capture are used to isolate EVs [90]. However, these methods often produce heterogeneous populations with varying sizes, cargo compositions, and surface markers, which complicates reproducibility and purity. Such variations can affect downstream applications, including therapeutic potency and safety [90]. A significant challenge is the co-isolation of non-vesicular particles and protein aggregates that may be inadvertently collected during isolation processes [91]. These by-products can introduce unwanted biological effects and contaminants into the final vesicle preparation, undermining the consistency expected from therapeutic products [91].

Several studies have highlighted these technical challenges. Comprehensive proteomic analyses of EV populations isolated using differential ultracentrifugation have demonstrated that this conventional method frequently co-isolates protein aggregates and non-vesicular contaminants [92]. Consequently, the resulting preparations are heterogeneous, with varying size distributions and cargo compositions, complicating reproducibility and purity in downstream applications. A study compared multiple isolation techniques, including ultracentrifugation, SEC, and immunoaffinity capture, and found that while SEC can reduce the level of soluble protein contaminants, it still yields EV fractions with variable cargo profiles that undermine standardisation [93]. Additionally, it was demonstrated that immunoaffinity capture methods, despite their ability to enrich for EV subpopulations with specific surface markers, may co-isolate non-vesicular particles through nonspecific interactions, reducing the reproducibility and purity of EV preparations [94].

#### 6.2.2. Scalability and Yield

Scalability and yield are significant challenges in the clinical-grade production of EVs. Current isolation protocols, like density gradient centrifugation, are time-consuming and labour-intensive, limiting scalability for large-scale manufacturing [95]. Syromiatnikova et al. (2022) noted that many conventional methods struggle to achieve high yields while maintaining the consistency and purity required for therapeutic applications [96]. Watson et al. (2016) demonstrated that conventional ultracentrifugation methods yield lower EV quantities and exhibit significant batch-to-batch variability [97].

It was reported that employing three-dimensional culture systems, such as hollow-fibre bioreactors, can enhance EV production by up to 40-fold compared to standard two-dimensional methods, offering a promising route toward scalable production [97]. Similarly, the development of standardised, high-throughput isolation methods to consistently yield sufficient quantities of EVs with uniform quality for therapeutic applications has been reported.

To address these limitations, the development of standardised, high-output extraction techniques is critical. Recent advancements in microfluidic devices and automated systems for EV isolation offer the potential to scale up production and improve yield, ensuring EV preparations meet the necessary quality and quantity standards for clinical use [84]. Additionally, optimizing membrane filtration and size exclusion chromatography techniques can enhance EV recovery while reducing processing time, which are essential in enabling the mass production of EV therapeutics [95].

#### 6.2.3. Characterisation and Quality Control

Characterising EVs involves the use of various techniques to define their size, morphology, and surface-marker profiles, but no single method has emerged as a universal standard. For instance, nanoparticle tracking analysis (NTA) is widely used to quantify EV size distributions and concentrations. However, a study highlighted that variations in sample dilution and measurement settings during NTA can lead to significant discrepancies in particle concentration measurements, emphasising the need for standardised protocols to ensure batch-to-batch consistency [98].

Similarly, electron microscopy, particularly cryo-electron microscopy (cryo-EM), provides high-resolution images that reveal subtle differences in EV morphology. Variations in sample fixation methods can significantly alter the apparent structural integrity of EVs, potentially affecting their biological activity [99].

High-resolution flow cytometry is also used to analyse the expression of EV surface markers, but its sensitivity is highly dependent on instrument settings and gating strategies.

#### 6.2.4. Storage and Stability

Optimising storage conditions is necessary to preserve EVs’ functional integrity. Studies have shown that storing EVs at −80 °C can preserve their stability and characteristics over time [100]. The choice of storage buffer has a significant impact on EV preservation as well. EV stability is enhanced by the use of phosphate-buffered saline supplemented with human serum albumin and trehalose (PBS-HAT) during several freeze–thaw cycles and extended storage at −80 °C.

EV integrity and therapeutic efficacy, on the other hand, might be jeopardised by poor storage settings, such as elevated temperatures or improper buffer compositions, which can result in particle loss, decreased purity, and fusion phenomena [101].

### 6.3. Ethical and Regulatory Considerations

The translation of EV-based therapies to clinical setting is accompanied by ethical and regulatory challenges:

#### 6.3.1. Safety and Efficacy

EV-based therapies show promise as modalities in regenerative medicine and drug delivery. However, translating these therapies to clinical use requires rigorous preclinical studies and well-structured clinical trials to evaluate safety and efficacy.

For example, a clinical trial assessed autologous dendritic cell-derived EVs loaded with MAGE tumour antigens in non-small cell lung cancer patients [100]. The EVs were well-tolerated, and an increase in natural killer cell activity was observed in nearly half of the participants. Despite these encouraging safety profiles, the trial did not achieve the desired therapeutic outcomes, highlighting the need for further investigation of efficacy parameters.

Preclinical studies have also raised potential concerns, such as off-target effects and unintended modulation of immune responses. While EVs facilitate intercellular communication and modulate immune responses, their immunostimulatory properties could lead to adverse effects if not properly controlled [102]. Additionally, understanding the long-term biodistribution of EVs is crucial.

#### 6.3.2. Source and Donor Considerations

Isolating EVs from donor cells or body fluids requires careful ethical considerations, particularly concerning donor consent and the choice between allogeneic and autologous sources. Obtaining informed consent from donors and securing approval from the relevant ethics committees were mandatory steps in the EV collection process [59]. They also highlight the necessity of screening human donors for infections before tissue collection to ensure safety in allogeneic applications.

The use of EVs derived from genetically modified cells adds another layer of complexity related to biosafety and public acceptance. While engineering EVs can enhance their targeting capabilities, these modifications raise concerns regarding safety profiles and the ethical implications of using genetically altered vesicles in therapeutic contexts [103].

#### 6.3.3. Regulatory Frameworks

The clinical translation of EV-based therapeutics is currently hindered by the lack of a unified regulatory framework. Regulatory agencies need to develop comprehensive guidelines covering manufacturing processes, quality control measures, and clinical testing protocols. These guidelines should include defining EV potency assays, ensuring product consistency, and addressing potential risks associated with bioactive cargo.

The International Society for Extracellular Vesicles has initiated efforts to create these guidelines through the Minimal Information for Studies of Extracellular Vesicles (MISEV) guidelines. These guidelines provide practical checklists for quantifying and characterising EVs to promote their clinical use [42]. Additionally, the U.S. Food and Drug Administration (FDA) has provided guidance for regulating gene and cell therapy products and has explored quality control strategies such as monitoring mesenchymal stem cell morphology during EV production to ensure the safety and efficacy of EV-based products.

Collaboration among researchers, industry stakeholders, and regulatory bodies is essential to establish clear standards and expedite the clinical translation of EV-based therapies [59].

#### 6.3.4. Cost and Accessibility

To ensure that EV-based treatments can be provided to a large number of patients, scalable and cost-effective production techniques must be developed. Various strategies have been explored to enhance EV yield and optimise production processes. For in-stance, changing cell culture conditions, such as using three-dimensional (3D) cultures, has been shown to significantly increase EV secretion. Cao et al. (2020) demonstrated a 19.4-fold increase in the protein concentration of small EVs, defined as vesicles with diameters typically less than 200 nm (often corresponding to MVs) [14], by employing a hollow-fibre bioreactor system for umbilical cord-derived mesenchymal stem cells (UC-MSCs) compared to conventional two-dimensional cultures [104].

In addition to optimizing culture conditions, the development of EV-mimetic nanovesicles offers a promising route for scalable production. Techniques like nitrogen cavitation, porous membrane extrusion, and sonication have been used to generate EV-like vesicles with homogeneous size and composition [96]. These methods not only reduce labour and time costs but also produce high yields of nanovesicles, potentially overcoming some limitations associated with natural EVs.

Furthermore, advancements in bioreactor technologies have enabled efficient large-scale production of EVs. For example, Cha et al. (2018) demonstrated that a simple and efficient 3D bioprocessing system significantly increased MV secretion from mesenchymal stem cells compared to conventional culture methods, highlighting the potential of bioreactor-based approaches in scaling up EV production [69].

## 7. Future Perspective

### 7.1. Potential Applications in Oral Health and Systemic Therapies

Research indicates that EVs from multiple sources can modulate the local immune environment while also suppressing pro-inflammatory cytokines and enhancing tissue regeneration at the same time, all within the oral cavity [105] EVs derived from mesenchymal stem cells are enriched with bioactive molecules that include cytokines, growth factors, and microRNAs, which shift immune cell phenotypes toward anti-inflammatory states and promote repair processes [106]. In several preclinical models, mesenchymal stem cell-derived EVs (MSC-EVs) downregulate TNF-α and other pro-inflammatory mediators while accelerating soft tissue healing. Hence, this suggests a potential mechanism that could be harnessed for oral tissue regeneration [105].

Similarly, studies on oral keratinocyte-derived exosomes indicate that EVs can stimulate re-epithelialisation and wound closure in the oral mucosa [107]. Their cargo, which includes signalling proteins and microRNAs, supports the restoration of tissue integrity and dampens excessive inflammatory responses at wound sites [106].

Additionally, plant-derived vesicles such as those isolated from ginger or tea leaves, have demonstrated that these natural nanovesicles carry anti-inflammatory and regenerative factors [1]. Their ability to modulate immune responses and promote tissue repair suggests they may have therapeutic potential in the oral cavity [106]. These findings support a growing consensus that EVs from MSCs, oral keratinocytes, and plant sources can act as cell-free therapeutic agents to modulate inflammation and promote tissue regeneration in oral applications [106,107].

EVs also have the potential to be effective precision drug delivery vehicles, with the potential to carry anti-inflammatory agents or regenerative signals specifically to periodontal pockets or sites of oral mucosal injury [58]. Their biocompatibility and ability to cross biological barriers allow these vesicles to be engineered for targeted delivery, increasing local therapeutic efficacy while reducing systemic side effects [58]. This approach is particularly promising for the management of periodontal disease and the promotion of the regeneration of oral mucosal tissues, as precise modulation of the local inflammatory microenvironment is critical for effective healing [58].

Engineered EVs may also be used in combination with biomaterials to promote craniofacial tissue regeneration [96]. When integrated into scaffold materials or hydrogels, engineered EVs can enhance the osteogenic differentiation of progenitor cells and facilitate the integration of new bone and soft tissue in craniofacial defects [1]. These findings suggest that EV-enhanced biomaterials represent a promising cell-free strategy for craniofacial tissue engineering and regeneration.

EVs are advantageous as drug delivery vehicles because they are biocompatible and can pass through a variety of biological barriers, including the blood–brain barrier [58]. Their phospholipid bilayer structure mimics that of cell membranes, which minimises immune recognition and allows them to circulate in the bloodstream [77]. This makes EVs attractive for systemic therapies, particularly for diseases that have an inflammatory component originating in the oral cavity.

For instance, chronic oral inflammation has been linked to the progression of neurodegenerative disorders such as Alzheimer’s disease. Oral pathogens like *Porphyromonas gingivalis* are associated with the triggering of neuroinflammation, which is a key factor in Alzheimer’s pathology [107]. By leveraging their ability to cross the blood–brain barrier, EVs can potentially be engineered to deliver anti-inflammatory or neuroprotective agents directly to the central nervous system. This could not only help to mitigate the inflammatory cascade initiated by oral dysbiosis but also slow down the progression of Alzheimer’s disease.

Moreover, EVs derived from mesenchymal stem cells have shown promise in preclinical studies by delivering cargo that modulates immune responses and supports neural repair [108]. Their capacity for targeted delivery highlights the potential to develop EV-based therapies that address both local oral inflammation and distant systemic effects, such as those observed in Alzheimer’s disease.

The therapeutic application of EV-based therapies and their mechanisms of action in oral inflammatory diseases are summarised in Table 5.

### 7.2. Emerging Technologies in EV Isolation, Engineering, and Characterisation

Technological advances are rapidly overcoming current challenges in EV production. For instance, novel microfluidic-based isolation techniques now allow for the separation of EVs from minimal sample volumes, with reduced processing times and minimised sample loss compared to traditional ultracentrifugation. Nanoparticle tracking analysis (NTA) can also provide more accurate and reproducible measurements of EV size distribution and concentration, which is important in standardising EV preparations across batches [60]. In addition, advancements in immunoaffinity capture techniques, where high-specificity antibodies target key EV surface markers, have enhanced the isolation of distinct EV subpopulations, increasing both the purity and scalability of the manufacturing process [95]. These innovations refine EV characterisation and quantification and pave the way for reproducible, scalable production protocols that meet stringent clinical-grade standards.

Recent studies have shown that bioengineering strategies are being developed to create synthetic EVs and to enhance natural EVs with targeted ligands, thereby improving tissue-specific delivery and controlled drug release. Engineered exosome mimetics have been developed to mimic the natural targeting properties of exosomes while overcoming limitations associated with natural EV yield and heterogeneity [109]. For instance, the surface of natural EVs was modified with targeting ligands using click chemistry, a highly efficient and biocompatible approach, to increase cellular uptake by specific target cells [109]. Moreover, artificial exosome-inspired vesicles constructed from liposomes have been engineered with receptor-targeting peptides and optimised lipid compositions to achieve prolonged circulation times and improved accumulation in diseased tissues [110]. By integrating precise surface modifications and artificial design principles, EV-based drug delivery systems could offer enhanced therapeutic efficacy with controlled drug release profiles and minimal adverse side effects.

### 7.3. Knowledge Gaps and Research Directions

Research on EVs has advanced, but there are still several obstacles to overcome. The absence of established procedures for EV isolation and characterisation is a key barrier that causes a great deal of variation in the final preparations. The yield, purity, and biological functionality of EV preparations produced by different techniques, including density gradient centrifugation, size-exclusion chromatography, differential ultracentrifugation, and polymer-based precipitation, vary [110]. This variability complicates comparisons between studies and poses a major barrier to the reproducible translation of preclinical findings into clinical applications.

For instance, the Minimal Information for Studies of Extracellular Vesicles 2023 (MISEV2023) guidelines published by the International Society for Extracellular Vesicles outline detailed recommendations for EV isolation, quantification, and characterisation. Despite the availability of these guidelines, their adoption remains inconsistent, and many studies still use non-standardised approaches [42]. Additionally, techniques used to characterise EVs, including nanoparticle tracking analysis, electron microscopy, and Western blotting for canonical markers, vary in sensitivity and specificity, contributing to the reproducibility issues in EV research [111].

More research is also needed to determine the optimal dosing regimens, biodistribution, and long-term safety of EV therapies. Several preclinical studies have successfully labelled and tracked EVs in vivo using advanced radiolabelling techniques; for instance, the non-invasive tracking of iodine-124-labelled MSC-EVs was demonstrated, revealing that EVs predominantly accumulate in organs such as the liver, spleen, lungs, and kidneys following intravenous administration [112].

These findings also underscore that EV biodistribution is variable and influenced by both the cell source and the route of administration [100]. Moreover, emerging strategies for the labelling and tracking of EVs have highlighted challenges in defining optimal dosing regimens, since the pharmacokinetic profiles of EVs appear to be dose-dependent [68]. Although short-term studies indicate a favourable safety profile for EV administration, long-term effects and the potential immunogenicity associated with repeated dosing remain largely unexplored [113]. These observations emphasise the critical need for increased and extended preclinical and clinical investigations to establish standardised dosing protocols and to further investigate the chronic pharmacokinetic behaviour and safety profile of EV-based therapies.

A deeper understanding of the molecular mechanisms of EV biogenesis, cargo selection, and cellular uptake is essential for the advancement of EV-based therapies for both oral and systemic diseases. EV biogenesis can occur via both ESCRT-dependent and ESCRT-independent pathways, with key roles for proteins such as TSG101, Alix, and various tetraspanins in mediating cargo sorting and vesicle formation [114].

Several studies have elucidated that recipient cell uptake of EVs occurs through diverse mechanisms, including clathrin-mediated endocytosis, phagocytosis, and direct membrane fusion, which direct the functional delivery of the vesicular cargo [115].

Investigation of these mechanisms is critical for a comprehensive understanding of EV biology, in addition to enabling researchers to fine tune EV-based therapeutic strategies by engineering the vesicle composition or modifying uptake pathways to enhance targeted delivery in both oral tissues and systemic applications [100].

Regulatory pathways must also evolve to support the clinical translation of EV-based therapies. In particular, robust and scalable production processes, such as 3D bioreactor systems and tangential flow filtration, that adhere to current good manufacturing practice (GMP) standards are essential for the production of EV preparations with consistent quality and potency [68]. Equally important is the development of clear regulatory approval frameworks that address the heterogeneity, stability, and mechanistic challenges unique to EVs, ensuring that they meet stringent safety and efficacy criteria [58].

Interdisciplinary collaboration among biologists, engineers, and clinicians will also be crucial to addressing these challenges and to accelerating the development of EV-based diagnostics and therapeutics.

## 8. Conclusions

**Figure 4:**
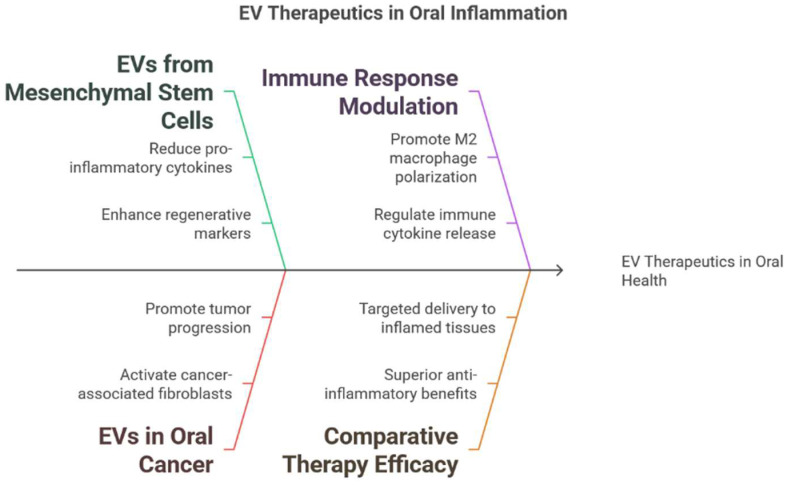
Therapeutic applications of EVs in oral inflammation. This figure summarises the therapeutic potential of mesenchymal stem cell-derived EVs, the role of EVs in immune response modulation, the implication of EVs in oral cancer, and the comparative efficacy of EV-based therapies.

Current evidence indicates that EVs are multifunctional mediators in the oral cavity, modulating inflammation, promoting tissue regeneration, and serving as diagnostic biomarkers. Preclinical studies have shown that MSC-derived EVs from dental pulp or gingival tissues reduce pro-inflammatory cytokines (e.g., TNF-α, IL-1β, and IL-6) [13,15] while enhancing regenerative factors that support angiogenesis and wound healing in periodontal defects. Similarly, exosomes from oral keratinocytes accelerate re-epithelialisation and wound closure via the transfer of functional microRNAs and proteins, suggesting their dual role in therapy and regeneration. In addition, plant-derived vesicles from ginger and tea leaves exhibit strong anti-inflammatory and tissue repair properties, further contributing to oral health. Together, these findings underscore the diverse origins and functions of EVs in the oral environment. EV-based therapies show promise for the management of oral inflammatory conditions. For instance, MSC-EVs have been demonstrated to modulate the local immune response and promote periodontal tissue regeneration in animal models, leading to reduced inflammatory cell infiltration and improved periodontal ligament integrity. Similarly, EVs have been used in preclinical models of oral mucositis to accelerate mucosal healing by enhancing epithelial cell proliferation and lowering pro-inflammatory cytokine levels [55]. Additionally, salivary EVs provide a non-invasive source of diagnostic biomarkers that reflect both local oral and systemic conditions [23], enabling early detection and personalised monitoring of diseases such as periodontitis, diabetes, and cardiovascular disorders. Overall, these findings highlight the potential for EV-based strategies to revolutionise both therapeutic and diagnostic approaches in oral inflammatory diseases (Figure 4).

## Figures and Tables

**Table 1:** Key components of the innate immune system.

Key Component of the Innate Immune System	Function	Citation
Saliva and Mucosal Barriers	Contain antimicrobial proteins like lysozyme and defensins; form a physical barrier against pathogens.	[23]
Neutrophils	First responders that migrate to infection sites, releasing enzymes and reactive oxygen species (ROS).	[24]
Macrophages and Dendritic Cells	Detect pathogens and trigger adaptive immunity by acting as antigen-presenting cells (APCs).	[20]
Toll-like Receptors (TLRs)	Identify microbiological components and initiate the synthesis of inflammatory cytokines.	[25]

**Table 2:** Key components of the adaptive immune system.

Key Components of the Adaptive Immune System	Function	Citation
CD4+ T-Helper Cells (Th1, Th2, Th17)	Regulate inflammation; Th1 promotes cell-mediated immunity, Th2 enhances humoral immunity, and Th17 recruits neutrophils.	[23]
B Cells and Antibodies	Produce antibodies (IgA, IgG, IgM) to neutralise pathogens and mediate the immune response.	[20]
Regulatory T Cells (Tregs)	Suppress an overabundance of immunological responses to avoid tissue harm.	[26]

**Table 3:** Therapeutic strategies of the immune system.

Therapeutic Strategy	Mechanism of Action	Citation
Anti-inflammatory Agents	NSAIDs, corticosteroids, and cytokine inhibitors reduce inflammation.	[26]
Host Modulation Therapy	Drugs like doxycycline and bisphosphonates regulate immune responses.	[20]
Probiotics and Prebiotics	Promote beneficial oral microbiota to maintain immune homeostasis.	[23]

**Table 4:** Sources of Extracellular Vesicles.

Source of EVs	Key Functions	Citation
Mesenchymal Stem Cell (MSC)-Derived EVs	Promote tissue repair, modulate immune responses, and reduce inflammation.	[19]
Platelet-Derived EVs	Enhance wound healing, stimulate fibroblast proliferation, and support vascular regeneration.	[35]
Immune Cell-Derived EVs	Modulate immune responses, alter cytokine production, and facilitate antigen presentation.	[36]
Epithelial Cell-Derived EVs	Regulate host–microbiome interactions and influence immune responses in oral health.	[28]
Stem Cell-Derived EVs from Other Sources	Exhibit regenerative properties similar to MSC-derived EVs but face ethical and production challenges.	[21]
Synthetic and Engineered EVs	Designed for targeted drug delivery, to carry therapeutic molecules, and enhance treatment efficacy.	[35]

**Table 5:** Key therapeutic applications and mechanisms of EV-based therapies in oral inflammatory diseases.

Oral Condition	EV Source	Mechanism of Action	Preclinical Studies
Periodontitis	Mesenchymal Stem Cell-derived EVs (MSC-EVs)	Modulation of the inflammatory microenvironment (e.g., downregulation of TNF-α and IL-6) and promotion of periodontal tissue regeneration.	Animal studies showing reduced inflammatory infiltration and improved periodontal ligament integrity.
Oral Mucositis	Oral Keratinocyte-derived Exosomes	Stimulation of re-epithelialisation and acceleration of mucosal wound closure.	Models demonstrating faster mucosal healing and reduced inflammatory cytokine levels.
Prevention of Malignant Transformation in Premalignant Lesions	EVs from various sources (MSC and keratinocytes)	Modulation of cell signalling pathways to suppress dysplastic progression.	Emerging experimental data suggesting EVs can help maintain normal cell behaviour in high-risk oral tissues.
Precision Drug Delivery in Periodontal Therapy	Engineered EVs from MSCs and other cells	Targeted delivery of anti-inflammatory or regenerative agents specifically to periodontal pockets.	Studies demonstrating enhanced local efficacy and reduced systemic side effects.
Craniofacial Tissue Regeneration	Engineered EVs combined with biomaterials	Promotion of osteogenic differentiation and integration of bone and soft tissue.	Preclinical models of craniofacial defects showing improved regeneration outcomes.
Systemic Therapy for Neurodegenerative Disorders	MSC-EVs engineered for CNS delivery	Ability to cross the blood–brain barrier and deliver neuroprotective agents, reducing neuroinflammation.	Preclinical studies linking oral inflammation with neurodegeneration and demonstrating targeted delivery in Alzheimer’s models.
Salivary EV Diagnostics	Salivary EVs	Non-invasive collection of EVs that contain diagnostic biomarkers (e.g., proteins and microRNAs) reflecting both oral and systemic health.	Studies correlating exosomal microRNA profiles with periodontitis severity and systemic conditions (diabetes and cardiovascular diseases).

## Data Availability

Not applicable.

## Abbreviations

The following abbreviations are used in this manuscript:

EVs	Extracellular vesicles
gDNA	Genomic DNA
sEV	Small extracellular vesicle
MVs	Microvesicles
bEVs	Bacterial extracellular vesicles
hPLT-EVs	Human platelet-derived extracellular vesicles
TLR	Toll-like receptor
NF-kB	Nuclear factor kappa B
RANKL	Nuclear factor kappa B ligand
NSAIDs	Nonsteroidal anti-inflammatory medications
TRAP	Tartrate-resistant acid phosphatase
OSCC	Oral squamous cell carcinoma
CAFs	Cancer-associated fibroblasts
GMSCs	Gingival mesenchymal stem cell-derived exosomes
PDLSCs	Periodontal ligament stem cells
MSCs	Mesenchymal stem cells
hPLT	Human platelet lysate
NTA	Nanoparticle tracking analysis
TEM	Transmission electron microscopy
HA	Hyaluronic acid
MHC	Major histocompatibility complex
RVG	Rabies virus glycoprotein
BBB	Blood–brain barrier
BM-MSCs	Bone marrow-derived mesenchymal stem cells
SHED	Human exfoliated deciduous teeth
VEGF	Vascular endothelial growth factor
SEC	Size-exclusion chromatography
PBS-HAT	Phosphate-buffered saline supplemented with human serum albumin and trehalose
FDA	Food and Drug Administration
MISEV	Minimal Information for Studies of Extracellular Vesicles
3D	Three-dimensional
UC-MSCs	Umbilical cord-derived mesenchymal stem cells
